# Oleocanthal Induces Mitochondrial Dysfunction in Breast Cancer Cell Lines Depending on c-MET Expression

**DOI:** 10.3390/antiox15040410

**Published:** 2026-03-25

**Authors:** Sergi Quetglas-Llobera, Pere Miquel Morla-Barcelo, Pilar Roca, Jorge Sastre-Serra, Mercedes Nadal-Serrano

**Affiliations:** 1 Grup Multidisciplinari d’Oncologia Translacional, Institut Universitari d’Investigació en Ciències de la Salut (IUNICS), Universitat de les Illes Balears (UIB), 07122 Palma, Spain; sergi.quetglas@uib.cat (S.Q.-L.); pere.morla@uib.es (P.M.M.-B.); pilar.roca@uib.es (P.R.); mercedes.nadal@uib.es (M.N.-S.); 2 Institut d’Investigació Sanitària de les Illes Balears (IdISBa), 07010 Palma, Spain; 3 Ciber Fisiopatología Obesidad y Nutrición (CB06/03), Instituto Salud Carlos III, 28029 Madrid, Spain

**Keywords:** breast cancer subtypes, oleocanthal, cancer metabolism, mitochondrial function, mitochondrial dynamics, c-MET signaling

## Abstract

Oleocanthal (OC), an anti-inflammatory and antioxidant phenolic compound exclusively found in extra virgin olive oil (EVOO), has emerged as a potential anticancer agent through multiple mechanisms of action, yet its impact on key processes such as cellular metabolism remains insufficiently characterized. Here, we investigated the metabolic and mitochondrial responses to OC across different breast cancer molecular subtypes. Triple-negative (MDA-MB-231) and luminal (MCF7, T47D) breast cancer cell lines were treated with OC to evaluate cell viability, cell cycle progression, metabolic enzyme expression, mitochondrial respiration, and mitochondrial network organization. OC responsiveness differed, being highest in MDA-MB-231 and lowest in T47D cells. Lactate dehydrogenase levels decreased in all cell lines, while mitochondrial response varied. MDA-MB-231 mitochondrial function was fully impaired, while MCF7 cells showed increased respiratory activity, with marked mitochondrial fragmentation, and T47D cells largely preserved mitochondrial integrity and function. Notably, the magnitude of OC effects correlated with *MET* expression, an established target of OC and a prognostic factor associated with reduced relapse-free survival within the triple-negative subtype. Collectively, these findings identify OC as a modulator of cancer cell metabolism and mitochondrial dynamics, with particular relevance in *MET*-high triple-negative breast cancers.

## 1. Introduction

Breast cancer remains the leading malignancy among women worldwide in terms of both incidence and mortality, and its global burden is expected to continue increasing in the coming years [1]. Importantly, this upward trend can be mitigated by addressing modifiable risk factors, as achieving higher quality-of-life scores leads to a reduced likelihood of developing this pathology [2]. Among these factors, diet has emerged as a major determinant influencing both the onset and progression of cancer. Unhealthy dietary patterns, such as the hypercaloric and high-fat Western diet, promote obesity, chronic inflammation and oxidative stress, thereby increasing the risk of breast malignancies [3,4,5]. In contrast, plant-based diets like the Mediterranean diet (MD), characterized by high intake of vegetables rich in micronutrients and bioactive compounds, moderate consumption of fish and dairy products, and low intake of processed red meat, are associated with a lower incidence of breast cancer [5,6].

In particular, extra virgin olive oil (EVOO) constitutes the primary fat source in the MD and is considered beneficial due to its high content of oleic acid, a monounsaturated fatty acid, as well as its content of over 200 bioactive compounds, such as terpenes, sterols, pigments and polyphenols [5,7]. An EVOO-enriched MD has been shown to reduce the breast cancer incidence compared with low-fat diets [8]. Notably, this protective effect appears to be specific to EVOO rather than refined olive oils [9]. This highlights the crucial role of its phenolic content, which is markedly diminished during the refining process [10]. Among the diverse phytochemicals present in EVOO, oleocanthal (OC) stands out for its remarkable health-related features. This phenolic secoiridoid has attracted considerable attention over the last years for its protective effects in various pathologies, including cancer, neurodegeneration, cardiovascular diseases and other inflammatory conditions [11,12,13]. It was first identified in 1993 by Montedoro et al. [14], but its biomedical relevance was not recognized until 2005, when Beauchamp et al. characterized it as a naturally occurring non-steroidal anti-inflammatory drug (NSAID), inhibiting cyclooxygenases (COX1/COX2) with greater potency than ibuprofen [15]. The same author also described OC as a potent antioxidant [16], a finding that was later corroborated [17], consistent with the overall antioxidant properties commonly attributed to EVOO.

OC was subsequently acknowledged in the oncology research field for its impact on the development of multiple cancer types like breast cancer [18,19,20], prostate cancer [20], hepatocellular cancer [21,22], colorectal cancer [21,23], gastric cancer [24] and melanoma [25,26]. The antitumoral effects of OC involve a broad spectrum of mechanisms targeting both cancer cells and the surrounding microenvironment [11]. These include inhibition of inflammation, induction of oxidative stress, destabilization of lysosomal membranes leading to apoptosis, regulation of epigenetic processes, reprogramming of the immune response, and inhibition of cancer-associated fibroblasts [11]. Remarkably, OC has been reported to suppress activation of the pro-oncogenic c-MET/STAT3 signaling axis [18,22,26]. c-MET, also known as the hepatocyte growth factor receptor (HGFR), belongs to the receptor tyrosine kinase (RTK) family and becomes activated upon binding to its ligand, hepatocyte growth factor (HGF), also referred to as scatter factor (SF). This interaction triggers multiple downstream signaling pathways including PI3K/Akt, STAT3 and the MAPK cascade. This receptor is known to play a role in normal breast development but also in cancer progression, regulating proliferation, migration, invasion and cell survival [27]. Emerging evidence further indicates that c-MET signaling could modulate cellular energetics, linking its inhibition to changes in mitochondrial dynamics [28,29].

In this context, while OC has been extensively studied for its general anti-tumoral effects, its direct impact on cancer cell metabolism and mitochondrial functionality remains poorly explored [21,30,31]. Metabolic reprogramming has long been established as a fundamental hallmark of cancer [32], enabling cancer cells to meet their high energetic and biosynthetic demands required to sustain uncontrolled growth under hostile environmental conditions. In this sense, the Warburg effect was the first described metabolic adaptation, reporting the predilection of tumoral cells towards aerobic glycolysis and imprecisely suggesting a mitochondrial disfunction. At present, the role of mitochondria in cancer cell biology has been extensively revisited, and their relevance has been acknowledged not only in terms of energy production but also in the regulation of intermediary metabolism, signal transduction and modulation of oxidative stress and redox homeostasis [33].

In this study, we aimed to investigate the not well understood impact of OC on breast cancer cell metabolism, with particular emphasis on mitochondrial function and dynamics. We also examined how these effects differed among breast cancer molecular subtypes and correlated with *MET gene* expression. Overall, these findings reveal that OC plays a role in modulating cancer cell metabolism and alters mitochondrial functionality in a cell-line-dependent manner.

## 2. Materials and Methods

### 2.1. Reagents

Oleocanthal (OC, SMB00810) was provided by Merck (St. Louis, MO, USA). Biowest (Riverside, MO, USA) supplied Dulbecco’s modified Eagle’s medium (DMEM) High Glucose and DMEM/F12. Biological Industries (Kibbutz Beit Haemek, Israel) supplied Fetal Bovine Serum (FBS), Horse Serum, antibiotics (penicillin and streptomycin). Capricorn Scientific (Ebsdorfergrund, Germany) supplied Dulbecco’s Phosphate-Buffered Saline (PBS). Routine chemicals were supplied by Merck (St. Louis, MO, USA) and Panreac (Barcelona, Spain).

### 2.2. Cell Culture

The human breast cancer cell lines MDA-MB-231 (triple-negative), MCF7 (luminal) and T47D (luminal) were obtained from the American Type Culture Collection (Manassas, VA, USA), while non-tumorigenic breast epithelial cell line MCF10A was kindly provided by Azucena Esparís-Ogando (IBSAL, Salamanca, Spain). Breast cancer cell lines were maintained in DMEM High Glucose supplemented with 10% (*v*/*v*) FBS and 1% (*v*/*v*) antibiotics. MCF10A cell line was maintained in DMEM/F12 supplemented with 20 ng/mL epidermal growth factor (STEMCELL Technologies, Vancouver, BC, Canada), 0.5 mg/mL hydrocortisone, 10 µg/mL insulin (Merck, St. Louis, MO, USA), 5% Horse Serum (*v*/*v*) and 1% (*v*/*v*) antibiotics. All cell lines were maintained at 37 °C in a 5% CO_2_ atmosphere.

### 2.3. Viability Assays

Cell viability was assessed by spectrophotometry using the crystal violet staining method, as previously described [34]. MDA-MB-231, MCF7 and T47D and MCF10A cells were seeded in 96-well plates at densities of 4.0 × 10^3^, 5.0 × 10^3^ and 6.0 × 10^3^ and 6.0 × 10^3^ cells/well, respectively, and allowed to adhere overnight. After 24 h, vehicle (0.375% DMSO) or increasing concentrations of OC (0–60 µM) were added for 48 h, maintaining the same DMSO concentration across all wells. Alternatively, cell viability was also assessed after 48 h of treatment with doxorubicin (0–10 µM), using PBS as vehicle.

### 2.4. Flow Cytometry

Cell cycle distribution was analyzed by flow cytometry following propidium iodide staining, as previously described with a minor modification [35]. Briefly, MDA-MB-231, MCF7 and T47D cells were seeded in 6-well plates at densities of 2.0 × 10^5^, 2.5 × 10^5^ and 3.0 × 10^5^ cells/well, respectively, and allowed to adhere overnight. After 24 h, the medium was replaced with fresh medium containing either vehicle (0.25% DMSO) or 40 µM OC. Following 48 h of OC treatment, cells were harvested, fixed in ice-cold 70% ethanol solution, and stored at −20 °C until analysis.

Prior to flow cytometry analysis, cells were washed and stained with a working solution containing 50 µg/mL propidium iodide (P4170, Merck, St. Louis, MO, USA) and 50 µg/mL RNase A (10109134001, Roche Diagnostics, Mannheim, Germany). After a 30 min incubation at room temperature and protected from light, the PI signal was quantified in 10,000 individual cells per sample using a BD FACSVerse™ Cell Analyzer from BD Biosciences (Franklin Lakes, NJ, USA). Excitation and emission wavelengths were set at 535 and 615 nm, respectively.

### 2.5. Western Blotting

MDA-MB-231, MCF7 and T47D cells were seeded in 6-well plates at densities of 2.0 × 10^5^, 2.5 × 10^5^ and 3.0 × 10^5^ cells/well, respectively, and allowed to adhere overnight. After 24 h, cells were treated with vehicle (0.25% DMSO) or 40 µM OC for 48 h. Samples were collected and lysed with RIPA buffer (50 mM Tris-HCl pH 7.5, 150 mM NaCl, 0.1% SDS, 0.5% deoxycholate, 1% Triton X-100, 1 mM EDTA) with HALT™ protease and phosphatase inhibitor from Thermo Fisher Scientific (Waltham, MA, USA), using a previously described protocol [36]. Protein content was determined with the Pierce™ BCA assay kit from Thermo Fisher Scientific (Waltham, MA, USA).

For Western Blots, 20 µg of protein were separated into SDS-PAGE gels and were electrotransferred onto nitrocellulose membranes with the Trans-blot^®^ Turbo™ Transfer System from Bio-Rad (Hercules, CA, USA). Membranes were blocked using a solution of 5% (*w*/*v*) non-fat powdered milk in TBS with 0.05% (*v*/*v*) Tween, for 1 h. The membranes were incubated overnight at 4 °C with the following antibodies: total OXPHOS antibody cocktail (ab110411, 1:1000) from Abcam (Orlando, FL, USA); LDHA (2012, 1:1000) and IDH2 (12652, 1:1000) from Cell Signaling Technology (Danvers, MA, USA); CS (1631-1-AP, 1:8000) from Proteintech (Rosemont, IL, USA); PDH-E1α (sc-377092, 1:1000) and GAPDH (sc-365062, 1:1000) from Santa Cruz Biotechnology (Santa Cruz, CA, USA). Protein bands were visualized using the Clarity™ Western ECL Substrate (Bio-Rad, Hercules, CA, USA) and chemiluminescent signals were detected with the ChemiDoc™ Imaging System (Bio-Rad, Hercules, CA, USA). The results were analyzed using the Image Lab Software (v6.1.0, Bio-Rad, Hercules, CA, USA) and GAPDH was used as a loading control for all blots.

### 2.6. Mitochondrial Function Assessment

Real-time oxygen consumption rates (OCRs) were determined using the Seahorse XFe96 Extracellular Flux Analyzer from Agilent (Santa Clara, CA, USA). MDA-MB-231, MCF7 and T47D cells were seeded at densities of 2.0 × 10^3^, 4.8 × 10^3^ and 9.6 × 10^3^ cells/well, respectively, into XFe96 well cell culture plates and incubated overnight to allow attachment, at 37 °C in a 5% CO_2_ atmosphere. After 24 h, cells were incubated with either vehicle (0.25% DMSO) or 40 µM OC. After 48 h of treatment, cells were maintained in 200 µL/well of XF assay media at 37 °C, in a non-CO_2_ incubator, for 1 h. During the incubation time, XF assay media solutions containing different mitochondrial complex inhibitors (1 µM oligomycin, 2 µM FCCP, 0.5 µM rotenone, and 0.5 µM antimycin A) were preloaded into the injection ports of the XFe96 sensor cartridge, for OCR measurements. Values were normalized to cell viability as measured with the crystal violet method (see Section 2.3).

### 2.7. Confocal Microscopy

MDA-MB-231, MCF7 and T47D cell lines were seeded on glass coverslips (72224-01, Electron Microscopy Sciences, Hatfield, PA, USA) inside 6-well plates at densities of 1.2 × 10^5^, 1.3 × 10^5^ and 1.8 × 10^5^ cells/well, respectively, and allowed to adhere overnight. After 24 h, cells were incubated with either vehicle (0.25% DMSO) or 40 µM OC for 48 h. Then, cells were incubated with 0.5 µM MitoTracker™ Green (M7514, Thermo Fisher Scientific, Waltham, MA, USA) for 1 h at 37 °C in a 5% CO_2_ atmosphere, protected from light. For DNA staining, 1 µg/mL Hoechst 33342 (B2261, Merck, St. Louis, MO, USA) was added in the last 5 min of incubation. The fluorescence was monitored with a Leica TCS-SPE Confocal Microscope, using a 63× immersion oil (1.47 N.A.) objective lens. Fluorescence excitation/emission wavelengths were set at 490/516 nm for MitoTracker™ Green and 350/455 nm for Hoechst 33342.

Mitochondrial morphological and network parameters were analyzed using the Mitochondria-Analyzer plugin for Fiji, designed by Ahsen Chaudhry at the University of British Columbia (2019). A tridimensional analysis was performed so that the plugin automatically processed all slices of the confocal images, thresholded the mitochondria, and calculated the corresponding parameters for each individual mitochondrion [37].

### 2.8. MET Gene Expression Analysis

The differential expression of the c-MET receptor gene (*MET*) in multiple breast cancer cell lines was assessed using data from the Cancer Dependency Map program (DepMap Public 25Q2 Dataset [38]. https://depmap.org/, accessed on 9 September 2025). Cell lines were classified into three different molecular subtype groups as follows: Luminal (ER+ and/or PR+, HER2+/−), HER2-enriched (ER−, PR−, HER2+) and triple-negative (ER−, PR−, HER2−). The complete panel of cell lines and the assigned molecular subtype are available in the Supplementary Table S1.

The distribution of breast cancer molecular subtypes in a cohort of breast cancer patients was determined in the low and high *MET* expression groups (divided by median) using data from the cBioPortal database (METABRIC Dataset [39,40]. https://www.cbioportal.org/, accessed on 7 October 2025). Molecular subtypes groups were defined as before.

### 2.9. Survival Analysis

Relapse-free survival (RFS) analyses of breast cancer patients with high or low *MET* expression (divided by median) were determined using the Kaplan–Meier plotter database [41] (https://kmplot.com/analysis/, accessed on 9 October 2025). The analysis was performed using data from the *203510_at* Affymetrix (Santa Clara, CA, USA) probe and separating patients according to the PAM50 gene expression-based classifier into four groups (Luminal A, Luminal B, HER2 and Basal). A LogRank *p*-value ≤ 0.05 was considered statistically significant.

### 2.10. Statistical Analysis

Statistical analyses were performed using the Statistical Program for the Social Sciences software (SPSS v27.0) from IBM (Armonk, NY, USA). Data obtained from cell lines is presented as mean ± standard error of the mean (SEM), and differences between vehicle- and OC-treated cells were assessed using a Student’s *t*-test. For human samples, the association between *MET gene* expression and breast cancer molecular subtype was evaluated using a chi-squared test. Statistical significance was set at a *p*-value ≤ 0.05 in all cases.

## 3. Results

### 3.1. Oleocanthal Reduces Cell Viability and Alters Cell Cycle Progression in Breast Cancer Cell Lines

**Figure 1:**
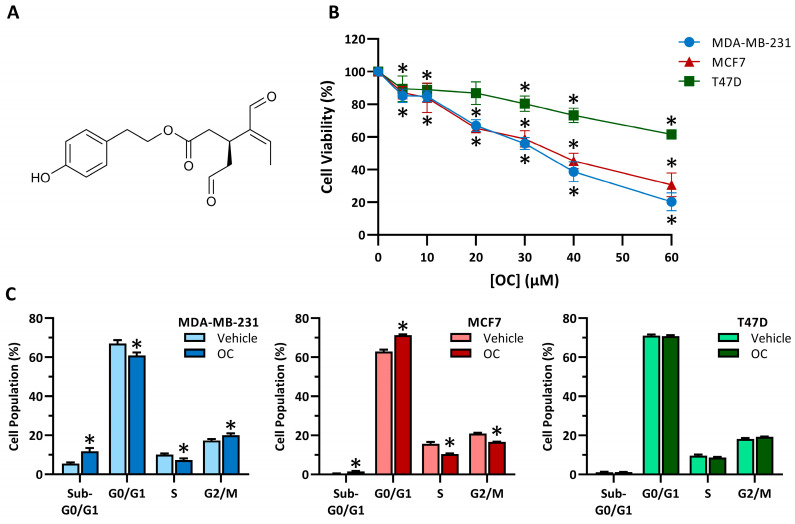
Oleocanthal reduces viability and alters cell cycle in breast cancer cell lines. (**A**) Molecular Structure of oleocanthal (OC). (**B**) Cell viability of MDA-MB-231 (●), MCF7 (▲) and T47D (■) breast cancer cell lines after treatment with increasing concentrations of oleocanthal (OC) for 48 h. Data are shown as means ± SEM (n = 3, biological replicates). Statistical differences between vehicle-treated cells and each concentration were determined using Student’s *t*-test (* *p* ≤ 0.05). (**C**) Cell cycle analysis of MDA-MB-231, MCF7 and T47D breast cancer cell lines after 48 h of OC treatment. Data are shown as means ± SEM (n = 3, technical replicates). Statistical differences between vehicle- and oleocanthal-treated cells were determined using Student’s *t*-test (* *p* ≤ 0.05).

To study the cytotoxic effect of oleocanthal (OC, Figure 1A) in breast cancer, we examined cell viability in lines of distinct molecular subtypes using a dose–response assay. MDA-MB-231 (triple-negative), MCF7 and T47D (luminal) cells were treated with increasing concentrations of OC, and cell viability was assessed (Figure 1B). The triple-negative cell line displayed a marked, concentration-dependent decrease in viability. Among luminal cell lines, MCF7 also showed a significant decrease in viability, whereas T47D cells were less sensitive, retaining approximately 60% viability at 60 µM. Non-tumorigenic breast epithelial MCF10A cells were also affected by OC (Supplementary Figure S1). For reference, we also assessed cell viability after treatment with doxorubicin in breast cancer cell lines (Supplementary Figure S2). In this case, all tumoral cell lines showed a rapid reduction in viability at low micromolar concentrations.

Based on viability and to further evaluate the impact of OC on cell proliferation, we analyzed the cell cycle distribution in cancer cell lines at 40 µM OC (Figure 1C). Consistent with the viability data, MDA-MB-231 cells were the most affected, with OC treatment inducing an accumulation of cells in the G2/M phase and a pronounced increase in the Sub-G0/G1 population. In contrast, MCF7 cells exhibited a distinct response, characterized by an increased proportion of cells in the G0/G1 phase and a significant, albeit modest, rise in the Sub-G0/G1 fraction. Alternatively, T47D cells did not show any significant changes.

### 3.2. Oleocanthal Induces Metabolic Rewiring and Disturbs Mitochondrial Function in Breast Cancer Cell Lines

**Figure 2:**
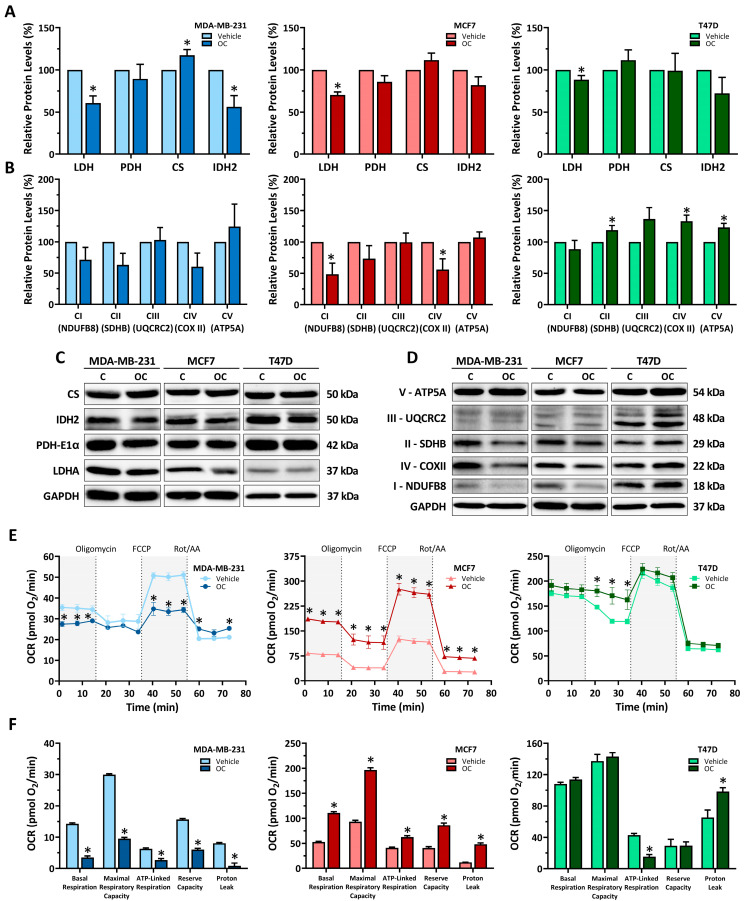
Oleocanthal promotes metabolic rewiring and modulates the mitochondrial respiration function in breast cancer cell lines. (**A**) Protein levels of metabolic enzymes (LDH, PDH, CS, IDH2) and (**B**) electron transport chain proteins of MDA-MB-231, MCF7 and T47D breast cancer cell lines after 48 h of oleocanthal (OC) treatment. (**C**,**D**) Representative bands of determined proteins. Data are shown as means ± SEM (n = 3, biological replicates) and vehicle-treated cells were set at 100% in each replicate. (**E**) Oxygen consumption rates after oligomycin, FCCP, and rotenone/antimycin A (Rot/AA) addition to MDA-MB-231, MCF7 and T47D breast cancer cell lines following 48 h of OC treatment. Data are shown as means ± SEM (n = 4, technical replicates). (**F**) Calculated parameters of Basal Respiration, Maximal Respiratory Capacity, ATP-Linked Respiration, Reserve Capacity and Proton Leak in MDA-MB-231, MCF7 and T47D breast cancer cell lines after 48 h of OC treatment. Data are shown as means ± SEM (n = 4, technical replicates). Statistical differences between vehicle- and oleocanthal-treated cells were determined using a Student’s *t*-test (* *p* ≤ 0.05) in all experiments.

After establishing the effects of OC on cell growth, we sought to explore its impact on cancer cell metabolism. As shown in Figure 2A,C, LDH protein levels decreased in all cell lines, whereas CS and IDH2 mitochondrial enzyme protein levels were only altered in MDA-MB-231 cells. Specifically, CS levels were increased, while IDH2 levels were reduced. We also determined the protein levels of representative subunits of each oxidative phosphorylation (OXPHOS) complex, as shown in Figure 2B,D. In this case, protein levels remained unaltered in MDA-MB-231 and changed in the luminal breast cancer cell lines. In MCF7, NDUFB8 and COXII showed decreased levels, while in T47D, SDHB, COXII and ATP5A presented increased levels.

Next, we evaluated mitochondrial function by measuring the oxygen consumption rate (OCR) and calculating the corresponding respiratory parameters (Figure 2E,F). The triple-negative MDA-MB-231 cell line exhibited the lowest basal OCR among the tested lines, and OC treatment caused a reduction in all assessed parameters, including basal respiration, maximal respiratory capacity, ATP-linked respiration, reserve capacity and proton leak. In contrast, luminal breast cancer cell lines showed either milder or opposite responses to OC. In MCF7 cells, all respiratory parameters were markedly elevated, whereas T47D displayed an increased proton leak at the expense of a nearly 75% decrease in the ATP-linked respiration.

### 3.3. Oleocanthal Alters Mitochondrial Network Dynamics in Breast Cancer Cell Lines

**Figure 3:**
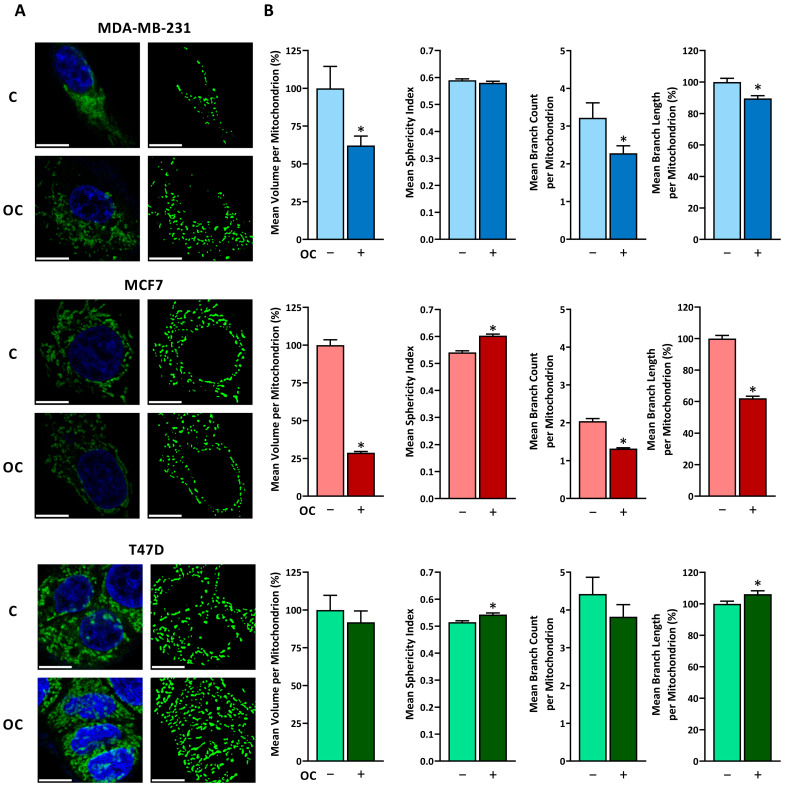
Oleocanthal induces alterations in the mitochondrial network in breast cancer cell lines. (**A**) Representative confocal microscopy images of mitochondria from MDA-MB-231, MCF7 and T47D breast cancer cell lines after 48 h of OC treatment. Left images show mitochondria stained with MitoTracker™ Green (green) and nuclei stained with Hoechst 33342 (blue). Right images show the thresholded mitochondrial network after applying the Mitochondria-Analyzer plugin from Fiji. Scale bar: 10 µm. (**B**) Calculated morphological (volume and sphericity) and network (branch count and branch length) parameters of individual mitochondrion using the Mitochondria-Analyzer plugin from Fiji. Data are shown as means ± SEM (n > 2000). Statistical differences between vehicle- and oleocanthal-treated cells were determined using a Student’s *t*-test (* *p* ≤ 0.05).

To gain further insight into the effects of OC on mitochondria, we analyzed confocal images of stained mitochondria after OC treatment (Figure 3A). The triple-negative MDA-MB-231 cells presented significantly smaller mitochondria after OC treatment (Figure 3B). A similar effect was observed in MCF7 cells, with mitochondria also becoming more spherical. In contrast, T47D cells maintained their mitochondrial volume, although they showed a slight increase in sphericity. Regarding network parameters, MDA-MB-231 and MCF7 mitochondria experienced a significant reduction in both branch number and length. Conversely, T47D mitochondria, which were the most highly branched under basal conditions, were mostly unaffected or slightly altered by OC treatment.

### 3.4. MET Expression Correlates with Oleocanthal Sensitivity in Breast Cancer Cell Lines and Predicts Poor Outcome in Basal-like Breast Cancer Patients

**Figure 4:**
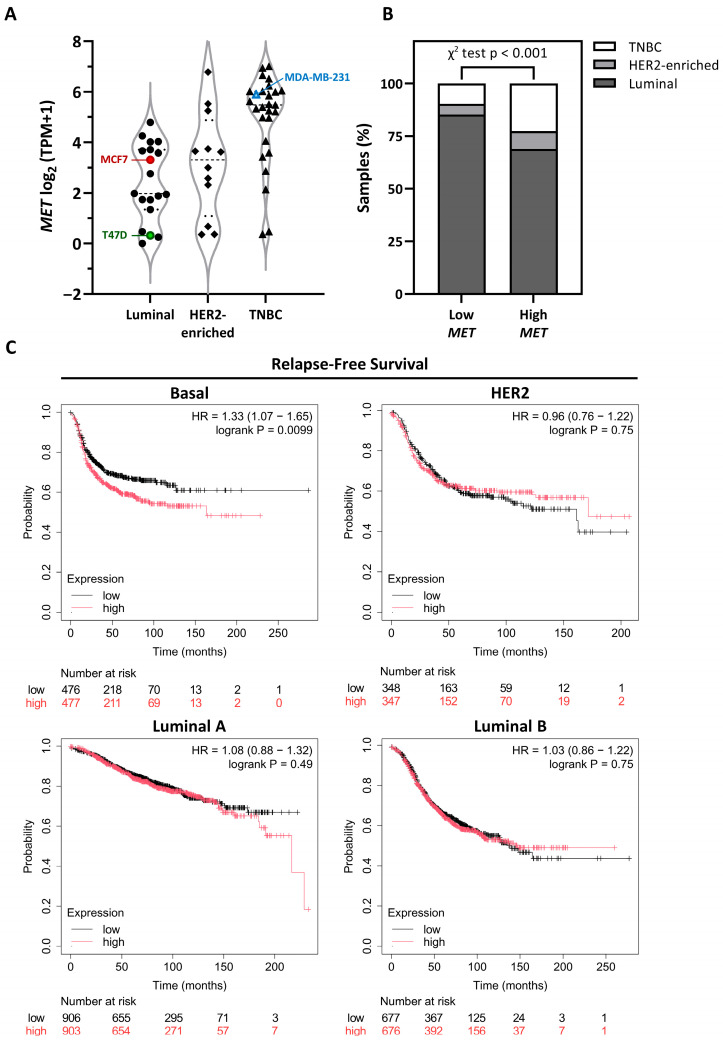
*MET* receptor overexpression is more frequent in triple-negative breast cancers and predicts poorer outcome in basal-like breast cancer patients. (**A**) Expression levels of *MET* mRNA (transcripts per million) in breast cancer cell lines of DepMap 25Q2 public database, classified by molecular subtype. (**B**) Distribution of breast cancer molecular subtypes among patients with low and high *MET* expression, divided by the median expression value (n = 990). Association between *MET* expression group and molecular subtype was assessed with a chi-squared test. Data was obtained from the cBioPortal METABRIC database. (**C**) Kaplan–Meier relapse-free survival analysis comparing patient groups of high (red) and low (black) expression of *MET*, divided by the median expression value. Data was obtained from the KMPlotter database.

To evaluate the potential relationship between *MET* expression and OC sensitivity, we analyzed a panel of breast cancer cell lines. As shown in Figure 4A, expression analyses revealed that MDA-MB-231 cells exhibited the highest *MET* expression, whereas T47D cells showed negligible expression. MCF7 cells displayed an intermediate expression level between the two. Overall, triple-negative breast cancer (TNBC) cell lines presented the highest *MET* expression levels, with most of them, including MDA-MB-231, showing greater expression than the maximum observed in luminal cell lines. Among luminal cell lines, T47D *MET* expression was one of the lowest, while in MCF7 it was moderate. HER2-enriched cell lines did not reveal any clear pattern, as *MET* expression ranged from nearly undetectable to very high across different cell lines.

We also investigated whether *MET* expression was associated with molecular subtypes in a cohort of breast cancer patients (Figure 4B). This association was found significant, as TNBC tumors were more likely to have a high *MET* expression, with their occurrence being doubled in this group. This increase was accompanied by a decrease in luminal breast cancer patients. In this cohort, HER2-enriched tumors represented the smallest fraction, and were slightly more frequent in the high *MET* expression group.

Finally, we assessed if the expression of *MET* receptor had any impact on the patient’s outcome. With this aim, relapse-free survival (RFS) was analyzed for high and low *MET* expression groups in a larger cohort, stratified following the PAM50 gene-based classifier. A significantly lower RFS was observed in the basal subtype with high *MET* expression, whereas no significant differences were detected in other molecular subtypes (Figure 4C).

## 4. Discussion

This study reveals a differential effect of OC on breast cancer cells survival, associated with impaired glycolytic activity and mitochondrial function. The magnitude of these effects is related to the expression of *MET*, a well-established molecular target of OC. Notably, triple-negative breast cancer (TNBC) cells, with the highest expression of *MET*, were the most sensitive.

First, we observed that OC reduced cell survival across all cell lines and differentially altered cell cycle dynamics, although this effect was noticeably milder in T47D cells. This aligns with previous studies that have extensively examined the antiproliferative activity of OC in various breast cancer models [18,19,20]. In particular, the TNBC cell line MDA-MB-231 is consistently reported as the most responsive, whereas the luminal cell lines, especially T47D, presented a lower sensitivity. These differences highlight the complexity of OC’s underlying mechanisms in different breast cancer subtypes. Indeed, OC has been shown to induce cell death via canonical apoptotic pathways, yet other caspase-independent mechanisms are also plausible and should be further considered to account for the observed variability in cellular responses [18,30].

To better contextualize the antiproliferative potency of OC and structurally related compounds, a recent work comprehensively evaluated the biological activity of six major secoiridoids naturally present in EVOO (oleocanthal, oleacein, oleuropein aglycone, ligstroside aglycone, oleomissional and oleocanthalic acid). In this study, OC exhibited the highest antitumoral effect across multiple cancer types, including breast cancer cell lines [42]. Moreover, combination assays involving two of these secoiridoids, and experiments using total phenolic extracts, identified OC as a major contributor to the observed reduction in cancer cell viability [43]. Although doxorubicin, a well-established DNA-intercalating chemotherapeutic agent, exhibited greater cytotoxicity than OC, the effects observed in our study highlight the promising anticancer potential of OC. Importantly, as one of the most effective polyphenols in EVOO, OC should be considered as a long-term nutraceutical in a dietary context rather than as a conventional chemotherapeutic agent.

Focusing on the specific effects of OC, the literature examination revealed that its potential role in tumor bioenergetics, currently recognized as a cornerstone of cancer biology [32,44], remains poorly characterized. In this study, a metabolic shift towards mitochondrial respiration was evidenced in all cell lines after OC treatment, reflected by a diminished glycolytic capacity. This observation is supported by previous findings showing that olive leaf extracts rich in oleuropein, direct precursor of oleocanthal, inhibited glycolysis in various cancer types, including the MDA-MB-231 cell line [45]. Such metabolic alteration could have important implications for tumor biology, particularly in TNBCs, which heavily rely on glycolytic metabolism [46,47].

To explore this metabolic shift in greater detail, we next focused on mitochondrial function and dynamics. As expected for the TNBC subtype, the MDA-MB-231 cells exhibited a markedly low OCR, and OC further diminished it. Analysis of their mitochondrial networks post-treatment revealed a more fragmented and less ramified morphology. Of note, TNBCs are characterized by a highly fissioned mitochondrial network which, under basal conditions, contributes to a more aggressive phenotype [48,49]. However, excessive fragmentation can trigger apoptosis and diminish their metastatic potential [49,50]. Conversely, luminal cells displayed higher OCRs, indicating their ability to retain energetically competent mitochondria [51]. Nonetheless, the two cell lines displayed substantially different responses following OC exposure. While MCF7 mitochondrial competence was severely compromised, T47D mitochondria were affected to a lesser extent. Importantly, dysfunctional mitochondria may serve as a primary source of reactive oxygen species (ROS), further contributing to cell damage [52]. In line with this, prior studies reported that OC-treated cancer cells presented depolarized mitochondrial membranes and elevated levels of ROS [21,24,30]. Although paradoxical, polyphenols can act as pro-oxidants in high doses and particular contexts like cancer [53,54], a phenomenon also described with other phenolic compounds [35,55]. Cancer cells maintain a delicate balance between ROS production, to drive proliferation and cell survival, and robust antioxidant defenses that mitigate ROS-induced damage [56]. Accordingly, the mitochondrial alterations we observed could disrupt this equilibrium, leading to cell death.

Regarding the specific differences observed in luminal breast cancer cell lines, these are likely driven by multiple underlying factors. In particular, previous studies have identified the estrogen receptor alpha-to-beta ratio (ERα/ERβ) as a determinant of cellular responses to cytotoxic agents in these breast cancer cell lines [57], with high ERβ expression in T47D cells contributing to the maintenance of a functional mitochondrial pool and a low oxidative stress state [58,59]. It is noteworthy that OC has also been recognized to target estrogen receptors, displaying higher affinity towards ERα than ERβ, and modulating estrogen signaling in a context-dependent manner [60,61]. In line with this, OC has been reported to reduce ERα expression in breast cancer cells [19]. Together, these observations may help explain why MCF7 cells, with a higher ERα/ERβ ratio, are more susceptible to OC-induced alterations than T47D cells, including mitochondrial perturbations. Paradoxically, recent evidence indicates that OC promotes mitochondrial health in colorectal cancer cells [31], underscoring the importance of considering cancer type-specific molecular contexts.

Another plausible contributor to the observed differential sensitivity is the expression of *MET*. The HGF/c-MET pair play a dynamic role during early embryogenesis and organogenesis [62], including breast tissue development. Specifically, stromal fibroblasts secrete HGF, while the mammary epithelial cells express the c-MET receptor, orchestrating the formation of mammary ducts through the branching of myoepithelial cells and proliferation of luminal cells [63,64]. However, breast carcinomas can obtain an aberrant c-MET signaling through a variety of mechanisms, including the acquisition of autocrine loops, promoting cancer progression [27]. In this context, OC has proven to effectively prevent c-MET phosphorylation [18,65], and therefore its activation, together with that of downstream effectors like STAT3, Akt, and MAP kinases [18,22,26,66]. Interestingly, T47D cells exhibited the lowest *MET* expression among the three cell lines, coinciding with their limited alterations following OC treatment. Alternatively, MCF7 and, especially, MDA-MB-231 displayed higher expression levels and were more sensitive to this phytochemical. Notably, triple-negative cell lines were the top *MET* expressers, in accordance with our clinical data analysis placing most TNBC tumors within the high-expression group. Moreover, high levels of this receptor had a clinical impact, with reduced relapse-free survival observed in patients with basal-like breast cancer, a subtype which largely overlaps with TNBC [67], but not in other molecular subtypes. This aligns with other studies linking elevated *MET* expression to poorer prognosis in different breast cancer subtypes, but particularly in TNBCs [68,69,70]. Mechanistically, c-MET can contribute to tumor development in this subtype through a variety of pathways. Of interest, c-MET has been reported to localize to the mitochondrial membrane, and its inhibition can lead to dysregulation of mitochondrial dynamics [28,29], consistent with the limited mitochondrial alterations observed in the *MET*-lowest T47D cells. Furthermore, c-MET can establish a positive feedback loop with HIF-1α [71,72], which is often highly active in TNBCs and drives its characteristic glycolytic dependance [73]. This interplay may underscore the potential impact of OC in targeting TNBCs, as its inhibitory effects on c-MET signaling may mitigate the glycolytic and aggressive phenotype of these tumors.

This study provides an original functional perspective of OC in breast cancer cell lines. Considering its pleiotropic effects, it is likely that additional mechanisms underlying its anticancer properties remain unknown. Thus, integrating omics approaches could help in the discovery of new OC targets [74,75]. From a translational perspective, an interesting characteristic of dietary phytochemicals is that, unlike traditional chemotherapeutic agents, they usually spare healthy cells from their harmful effects. In our experimental conditions, however, we observed a marked reduction in the viability of MCF10A breast epithelial cells following OC treatment. This contrasts with previous studies reporting little or no effect in this cell line [18], as well as in other non-tumorigenic models, including primary hepatocytes [21], neonatal human dermal fibroblasts [24] or embryonic kidney cells [30]. This discrepancy requires further investigation to clarify which underlying factors, such as vehicle-related baseline stress, influence MCF10A cell sensitivity to OC. Additionally, other important aspects should be taken into account. First, the health benefits associated with the Mediterranean diet, particularly EVOO consumption, cannot be attributed exclusively to OC, but rather to the combined effects of multiple bioactive compounds [5]. Second, the in vitro concentrations used in the present study are unlikely to be achieved through a typical dietary intake. This is due to several factors, including the substantial variability in oleocanthal content among different olive oils, which can range from negligible amounts to over 3 mM [11], as well as its low oral bioavailability, resulting from both poor absorption and rapid metabolism [76,77,78]. Nevertheless, a pilot study (NCT04215367) reported clinical improvements in chronic lymphocytic leukemia patients following a dietary intervention with OC-rich EVOO, whereas no such effects were observed with OC-poor EVOO [79]. These findings suggest that dietary intervention may still represent a feasible approach for OC to exert biologically relevant effects in vivo. Furthermore, the development of OC analogs with improved pharmacokinetic properties [80], together with OC-based formulations that would allow its direct oral administration [81,82], may help overcome current translational limitations in the following years.

## 5. Conclusions

Altogether, we provide a novel mode of action for oleocanthal as a metabolic and mitochondrial modulator, highlighting its differential effects across breast cancer molecular subtypes. With the current available information, we support the potential use of oleocanthal in a nutraceutical-based approach for breast cancer management, particularly in triple-negative tumors, considering their high *MET* expression and glycolytic dependance.

## Data Availability

The original contributions presented in this study are included in the article/Supplementary Material. Further inquiries can be directed to the corresponding author.

## Supplementary Materials

The following supporting information can be downloaded at: https://www.mdpi.com/article/10.3390/antiox15040410/s1, Table S1: *MET* expression levels (transcripts per million) and molecular subtype classification of breast cancer cell lines; Figure S1: Effect of oleocanthal on breast epithelial MCF10A cells viability; Figure S2: Effect of doxorubicin on breast cancer cells viability.

## Abbreviations

The following abbreviations are used in this manuscript:

ATP5A	ATP Synthase F_1_ Subunit A
COX1	Cyclooxygenase 1
COX2	Cyclooxygenase 2
COXII	Cytochrome c Oxidase Subunit II
CS	Citrate Synthase
DMEM	Dulbecco’s Modified Eagle Medium
ER	Estrogen Receptor
EVOO	Extra Virgin Olive Oil
FBS	Fetal Bovine Serum
HER2	Human Epidermal Growth Factor Receptor 2
HGF/SF	Hepatocyte Growth Factor/Scatter Factor
HGFR	Hepatocyte Growth Factor Receptor
HIF-1α	Hypoxia-Inducible Factor 1-alpha
IDH2	Isocitrate Dehydrogenase 2
LDH	Lactate Dehydrogenase
MAPK	Mitogen-Activated Protein Kinase
MD	Mediterranean Diet
NDUFB8	NADH Dehydrogenase [Ubiquinone] Subunit B8
NF-κB	Nuclear factor kappa B
NSAID	Non-Steroidal Anti-Inflammatory Drug
OC	Oleocanthal
OCR	Oxygen Consumption Rate
OXPHOS	Oxidative Phosphorylation
PBS	Phosphate-Buffered Saline
PDH	Pyruvate Dehydrogenase
PI3K	Phosphoinositide-3-Kinase
PR	Progesterone Receptor
RFS	Relapse-Free Survival
ROS	Reactive Oxygen Species
RTK	Receptor Tyrosine Kinase
SDHB	Succinate Dehydrogenase Subunit B
STAT3	Signal Transducer and Activator of Transcription 3
TNBC	Triple-Negative Breast Cancer
UQCRC2	Ubiquinol–Cytochrome c Reductase Core Protein 2
