# Supplementary material for: Oleocanthal Induces Mitochondrial Dysfunction in Breast Cancer Cell Lines Depending on c-MET Expression
Source: Antioxidants (Basel). 2026 Mar 25;15(4):410. doi: 10.3390/antiox15040410 (PMC13113521; doi:10.3390/antiox15040410)
Supplement: Supplementary file 1 [file antioxidants-15-00410-s001.zip › antioxidants-4149105-supplementary.pdf]

Table S1. *MET* expression levels (transcripts per million) and molecular subtype classification of breast cancer cell lines.

Cell Line	*MET* log2 (TPM+1)	Molecular Subtype	Cell Line	*MET* log2 (TPM+1)	Molecular Subtype
BCK4	0.0000	Luminal	JIMT1	5.5236	HER2-enriched
ZR7530	0.2448	Luminal	HCC1954	6.7824	HER2-enriched
T47D	0.3162	Luminal	SNU2372	0.3578	TNBC
CAMA1	0.4647	Luminal	DU4475	0.4682	TNBC
MDAMB134VI	1.3397	Luminal	HCC1599	2.1322	TNBC
SUM44PE	1.7321	Luminal	HCC2157	2.8607	TNBC
MDAMB175VII	1.7400	Luminal	SUM149PT	3.4136	TNBC
EFM19	1.8771	Luminal	SUM102PT	3.5864	TNBC
UACC3133	1.9423	Luminal	MDAMB157	4.0538	TNBC
MDAMB415	1.9771	Luminal	CAL120	4.9600	TNBC
ZR751	2.7587	Luminal	MDAMB436	4.9739	TNBC
MCF7	3.3073	Luminal	MDAMB468	5.2067	TNBC
EFM192A	3.5789	Luminal	HCC1395	5.2517	TNBC
BT474	3.6612	Luminal	HCC1806	5.3322	TNBC
HCC1500	3.7119	Luminal	SUM1315MO2	5.3955	TNBC
BT483	4.0172	Luminal	CAL51	5.4781	TNBC
HCC1428	4.0288	Luminal	HCC1187	5.5026	TNBC
KPL1	4.2581	Luminal	BT549	5.6187	TNBC
MDAMB361	4.7937	Luminal	HCC1143	5.8441	TNBC
HCC1419	0.3568	HER2-enriched	MDAMB231	5.8987	TNBC
HCC2218	0.3600	HER2-enriched	BT20	5.9941	TNBC
UACC812	0.6742	HER2-enriched	SUM159PT	6.0124	TNBC
HCC202	2.3143	HER2-enriched	HCC1937	6.0124	TNBC
UACC3199	2.5786	HER2-enriched	CAL851	6.0433	TNBC
SKBR3	3.0008	HER2-enriched	HCC70	6.2388	TNBC
SUM190PT	3.6225	HER2-enriched	HS578T	6.5151	TNBC
UACC893	3.6517	HER2-enriched	SUM229PE	6.6546	TNBC
HCC1569	3.7428	HER2-enriched	HDQP1	6.9345	TNBC
AU565	5.2526	HER2-enriched	HCC38	6.9986	TNBC

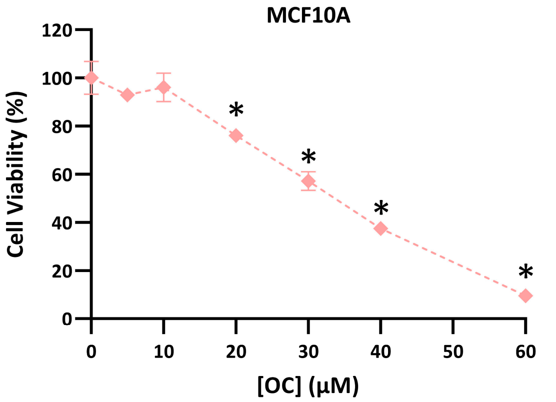

Figure S1. Effect of oleocanthal on breast epithelial MCF10A cells viability. Cell viability of MCF10A cell line after treatment with increasing concentrations of oleocanthal (OC) for 48 h. Data are shown as means ± SEM (n=3, technical replicates). Statistical differences between vehicle-treated cells and each concentration were determined using Student's t-test (* $p \leq 0.05$).

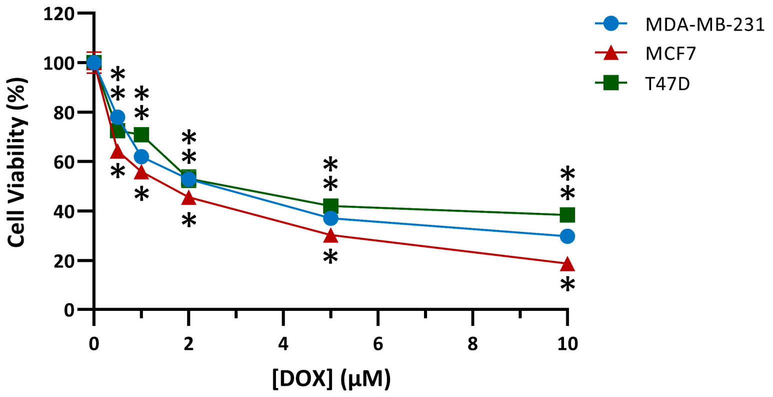

Figure S2. Effect of doxorubicin on breast cancer cells viability. Cell viability of MDA-MB-231 (●), MCF7 (▲) and T47D (■) breast cancer cell lines after treatment with increasing concentrations of doxorubicin (DOX) for 48 h. Data are shown as means ± SEM (n=3, technical replicates). Statistical differences between vehicle-treated cells and each concentration were determined using Student's t-test (* $p \leq 0.05$).
